# Sustained Impairment in Cardiopulmonary Exercise Capacity Testing in Patients after COVID-19: A Single Center Experience

**DOI:** 10.1155/2022/2466789

**Published:** 2022-03-01

**Authors:** Georg Evers, Arik Bernard Schulze, Irina Osiaevi, Kimon Harmening, Richard Vollenberg, Rainer Wiewrodt, Rudin Pistulli, Matthias Boentert, Phil-Robin Tepasse, Juergen R. Sindermann, Ali Yilmaz, Michael Mohr

**Affiliations:** ^1^Department of Medicine A,Hematology,Oncology and Pulmonary Medicine, University Hospital Münster, Münster, Germany; ^2^Department of Medicine B for Gastroenterology,Hepatology,Endocrinology and Clinical Infectiology, University Hospital Münster, Münster, Germany; ^3^Department of Cardiology I,Coronary and Peripheral Vascular Disease and Heart Failure, University Hospital Münster, Münster, Germany; ^4^Department of Neurology, University Hospital Münster, Münster, Germany

## Abstract

**Background:**

Following COVID-19, patients often present with ongoing symptoms comparable to chronic fatigue and subjective deterioration of exercise capacity (EC), which has been recently described as postacute COVID-19 syndrome.

**Objective:**

To objectify the reduced EC after COVID-19 and to evaluate for pathologic limitations.

**Methods:**

Thirty patients with subjective limitation of EC performed cardiopulmonary exercise testing (CPET). If objectively limited in EC or deteriorated in oxygen pulse, we offered cardiac stress magnetic resonance imaging (MRI) and a follow-up CPET.

**Results:**

Eighteen male and 12 female patients were included. Limited relative EC was detected in 11/30 (36.7%) patients. Limitation correlated with reduced body weight-indexed peak oxygen (O_2_) uptake (peakV̇O_2_/kg) (mean 74.7 (±7.1) % vs. 103.6 (±14.9) %, *p* < 0.001). Reduced peakV̇O_2_/kg was found in 18/30 (60.0%) patients with limited EC. Patients with reduced EC widely presented an impaired maximum O_2_ pulse (75.7% (±5.6) vs. 106.8% (±13.9), *p* < 0.001). Abnormal gas exchange was absent in all limited EC patients. Moreover, no patient showed signs of reduced pulmonary perfusion. Using cardiac MRI, diminished biventricular ejection fraction was ruled out in 16 patients as a possible cause for reduced O_2_ pulse. Despite noncontrolled training exercises, follow-up CPET did not reveal any exercise improvements.

**Conclusions:**

Deterioration of EC was not associated with ventilatory or pulmonary vascular limitation. Exercise limitation was related to both reduced O_2_ pulse and peakV̇O_2_/kg, which, however, did not correlate with the initial severity of COVID-19. We hypothesize that impaired microcirculation or limited peripheral O_2_ utilization might be causative for prolonged deterioration of EC following acute COVID-19 infection.

## 1. Introduction

In late 2019, a novel SARS-associated coronavirus was identified as the cause of an increased incidence of pneumonia cases in Wuhan, Hubei Province of China [1]. In early 2020, the World Health Organization designated the disease as coronavirus disease 2019 (COVID-19) [2], caused by the enveloped, single-stranded RNA virus “severe acute respiratory syndrome coronavirus 2” (SARS-CoV-2). Ever since, the disease has developed into a global pandemic. Acute infection clinically presents from mild [3] to severe and critical forms. Severe forms included respiratory distress (respiratory rate ≥30/min) and/or oxygen saturation (SpO_2_) ≤ 93% at ambient air during rest. Moreover, critical courses were in need of ventilation and/or extracorporeal membrane oxygenation [4, 5].

Due to the global spread of coronavirus disease 2019 (COVID-19), the number of individuals recovering from acute SARS-CoV-2 infection is on the increase. A growing number of observational data suggest that patients may suffer from a wide range of symptoms after recovery from acute illness referred to by several terms such as “long COVID” or “postacute COVID-19 syndrome” [6–12]. However, postacute illness and persistent symptoms following COVID-19 show similar features of recovery from other viral diseases or sepsis [13–16].

After the acute and infectious COVID-19 phase, a so-called “long COVID syndrome” is thought to begin 3 to 4 weeks after the onset of acute symptoms in more than one-third of the patients [17, 18]. This syndrome manifests either as an acute thromboembolic complication with concomitant deterioration or as a long-term persistent symptom complex with no currently known clinical correlation [19, 20]. So far, fatigue and dyspnea have been mentioned as the most common symptoms in several observational studies [17–20]. The underlying causes of long COVID syndrome are not fully understood. Currently, it appears that several factors might be involved, such as cardiac sequelae, impairment of pulmonary blood flow and gas exchange, or restricted lung function [21].

To gain insight into the unspecific form of long COVID syndrome and to further analyze the pathophysiological background of persistent fatigue and dyspnea, we performed pulmonary function tests followed by bicycle-exhausting cardiopulmonary exercise testing (CPET) to evaluate for physical limitations caused by pulmonary, pulmonary vascular, cardiocirculatory, or gas exchange pathologies in patients with subjective deterioration in exercise capacity (EC) after COVID-19.

## 2. Methods

After approval of the study conducted by the local ethics committee (Ref. 2020-585-f-S), patients with persistent, subjective deterioration in EC or with symptoms consistent with dyspnea or fatigue at follow-up visits after COVID-19 were offered prospective follow-up bicycle-exhausting cardiopulmonary exercise testing (CPET) at our outpatient clinic. Each patient suffered from infection with the nonvariant SARS-CoV-2. According to the current World Health Organization guidance criteria [3–5], patients were categorized into mild/moderate disease, if pure outpatient treatment was performed. Severe illness was defined by inpatient care and/or oxygen supplementation. Critical illness included noninvasive and invasive ventilation due to acute respiratory failure.

As per protocol, we documented the modified medical research council (mMRC) severity of breathlessness score and evaluated spirometry and/or body plethysmography (JAEGER® MasterScreen Body, CareFusion Germany 234 GmbH, 97204 Höchberg, Germany, and SentrySuite, Vers-No. 2.19.4, CareFusion Germany 234 GmbH, 97204 Höchberg, Germany) before first bicycle CPET evaluation (CareFusion Type MasterScreen CPX and CareFusion Germany 234 GmbH, 97204 Höchberg, Germany). Every patient gave informed consent before CPET. Spirometry and/or body plethysmography were performed according to the harmonized European Respiratory Society (ERS) and American Thoracic Society (ATS) guidelines [22].

Exhausting CPET was performed via bicycle test (GE Healthcare ergometer eBike basic, GE Healthcare Germany, 79111 Freiburg, Germany), with baseline resistance (5; 10; 12.5; 25; 50 watts (W)) and individual, continuous increase in resistance by 5, 10, 12, 25, or 50 W over two minutes prior to CPET to best achieve exhaustion criteria within 12 minutes of exercise. Arterialized capillary blood gas analyses were sampled during CPET. Wasserman plots and raw data were documented.

Cessation criteria for CPET included respiratory exchange ratio (RER) ≥ 1.25, signs of ischemia or ventricular arrhythmia in the electrocardiogram, dizziness, cold sweat, and/or vertigo, unacceptable subjective shortness of breath or chest pain, a desaturation below 80%, muscular exhaustion, a systolic blood pressure >250 mmHg, a diastolic blood pressure >120 mmHg, and a clinically relevant blood pressure decrease (−20 mmHg) while at increasing resistance [23].

Physical limitation in exhausting CPET was considered a workload (watt) < 100% of age-, weight-, and size-adjusted required range. Standard values in CPET for all evaluated and predicted values included calculations based on Gläser et al. [24].

When indicated and consented, patients underwent cardiac magnetic resonance imaging (cMRI) after documentation of possible cardiocirculatory CPET limitation *(i.e.,* deteriorated oxygen pulse, early oxygen pulse plateau, insufficient heart rate increase, abnormalities in electrocardiogram during EC, and blood pressure decline during EC).

After exclusion of organ function limitations, patients were recommended to increase exercise activities such as physical strength and endurance. Professional medical rehabilitation was not performed in any of the patients during the period of evaluation. Every patient was offered a CPET follow-up evaluation. Follow-up CPET was performed, if agreed and either objectively limited in EC, limited in oxygen pulse, or if still suffering from extensive subjective exercise limitation.

To describe the cohort, we used mean, standard deviation (SD), raw count, and frequencies. Twofold associations between categorical variables were analyzed via Fisher's exact test or chi-square test, if applicable. Continuous and ordinal variables were tested using either unpaired *t*-test or Mann-Whitney *U*-test depending on the normality of the data. As all measured and predicted values revealed a normal distribution, related variables between first and second CPET were analyzed via paired *t*-test.

Data collection and calculations and graphs were performed using IBM® SPSS® Statistics Version 27 (released 2020, IBM Corp., Armonk, NY, USA). The local significance level was set to 0.05. Due to the explorative character of the analysis, an adjustment to multiplicity was not determined.

## 3. Results

Baseline characteristics of the cohort can be found in Table 1. We included multiple CPET analyses of 18 male patients and 12 female patients, aged 51.5 (±14.1) years at COVID-19 diagnosis, with 21 patients undergoing inpatient care. The average time from COVID-19 diagnosis to first CPET was 4.3 months.

Objective limitation in EC was present in 11/30 (36.6%) patients during the first and in 12/16 (75%) patients during the second CPET. In the observed cohort, all documented values and differences were normally distributed. Time from SARS-CoV-2-positive polymerase chain reaction testing to first CPET (CPET1) was 4.7 (±2.1) months in the nonlimited cohort and 3.6 (±1.4) months in the limited cohort (*t*-test *p*=0.079), and another 3.5 (±1.1) months in the nonlimited cohort and 3.4 (±1.4) months in the limited cohort to follow-up CPET (CPET2) (*t*-test *p*=0.943). In both cases, differences were not statistically significant.

Regarding CPET1, to further assess the cause of EC limitation, we first correlated non-CPET values between the subcohorts (Table 2). Here, neither gender nor body mass index significantly differed in distribution. Still, 63.3% of the patients revealed a body mass index (BMI) > 25 kg/m^2^ and most of the analyzed patients were men. However, with a mean BMI of 27 kg/m^2^, average weight and size distribution resulted in moderately increased overweight. Moreover, obesity was present in only 4 patients (1st-degree obesity in 3 patients and 3rd-degree obesity in one patient). Finally, there were no statistically significant differences in terms of BMI distribution (BMI ≤25 kg/m^2^ vs. BMI 25,1–30,0 kg/m^2^ vs. BMI >30,0 kg/m^2^, Mann-Whitney *Up*=0.064). However, with respect to age, the borderline significant occurrence of younger patients in the limited patient cohort was observed (*p*=0.05). Moreover, ventilatory parameters such as forced expiratory volume in one second (FEV1) and forced vital capacity (FVC) and subjective dyspnea grade, measured by modified medical research council (mMRC) scale, did not differ between nonlimited and limited individuals. Likewise, inpatient or outpatient care and COVID-19 severity presented in equal distribution between the two cohorts.

Following this, we analyzed relevant CPET parameters between the cohorts (Table 3). With respect to gas exchange at rest and during exercise, no significant differences of oxygen partial pressure (pO_2_) and carbon dioxide partial pressure (pCO_2_) at rest could be documented between limited patients and nonlimited ones. However, with respect to exhaustion, gas exchange was the exercise-limiting factor for nonlimited patients, resulting in higher alveolar-arterial pressure gradients in comparison to the limited subjects (*p*=0.006). Yet, gas exchange was not a limiting factor in limited individuals with low workload (*i.e.,* <100%) and low body weight-indexed oxygen uptake (alveolar-arterial oxygen gradient (AaDO_2_) mean 29.4 mmHg). Here, hypoxemia and hypercapnia could be excluded. Nevertheless, subjective dyspnea was equally prominent in both cohorts (*c.f.,*Table 2).

In association with reduced EC, body weight-indexed oxygen uptake (peak V̇O_2_/kg) was found to be 74.7 (±7.1) % in limited individuals and 103.6 (±14.9) % in nonlimited ones (*p* < 0.001). With respect to the cause of EC limitation, we found an insufficient O_2_ pulse increment, defined as maximum O_2_ pulse <84% of predicted normal range, in 12/30 (40%) participants within the first CPET performance (Fisher's exact test *p* < 0.001, data not shown). The average O_2_ pulse was 75.7 (±5.6) % in limited patients and 106.7 (±13.9) % in nonlimited patients (*t*-test *p* < 0.001), offering an indicator for cardiocirculatory limitation (*c.f.,*Table 3).

During the CPET phase of increasing resistance, nonlimited patients presented a better increment of their respiratory minute volume (V̇E) and fully used their breathing reserve. Yet, as mentioned before, in limited patients gas exchange impairment was not evident. Of further interest, none of the patients studied showed evidence of persistent pulmonary vascular disease (*e.g.,* elevated respiratory equivalents).

**Figure 1:**
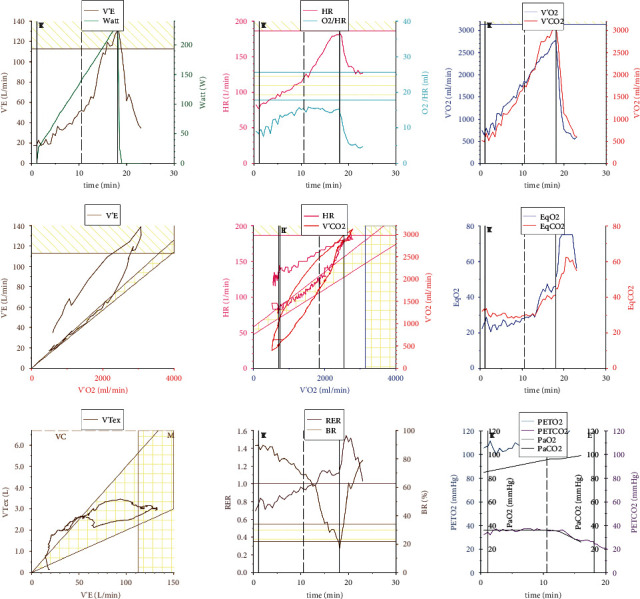
Representative 9-panel display of Wasserman of a limited post-COVID-19 patient. V'E: respiratory minute volume, HR: heart rate, O_2_/HR: oxygen pulse, V'O_2_: oxygen uptake capacity, V'CO_2_: carbon dioxide elimination capacity/carbon dioxide output, EqO_2_: oxygen ventilatory equivalents, EqCO_2_: carbon dioxide ventilatory equivalents, VTex: expiratory tidal volume, RER: respiratory exchange rate, BR: breathing reserve, PETO_2_: end-tidal partial pressure of oxygen, PETCO_2_: end-tidal partial pressure of carbon dioxide, PaO_2_: arterial/arterialized capillary oxygen partial pressure, and PaCO_2_: arterial/arterialized capillary carbon dioxide partial pressure.

Taken together, the presented data deduce a typical CPET pathology in post-COVID-19 patients with an insufficient O_2_ pulse increment and reduced oxygen uptake capacity, hinting at a cardiocirculatory EC limitation. A representative 9-panel display of Wasserman of a CPET analysis is shown in Figure 1. Here, striking features were the reduced oxygen uptake capacity (panel 3) and the insufficient O_2_ pulse increase (panel 2) during the exercise phase. Features of pulmonary(-vascular) limitations (panel 4 and 6) or gas exchange disorders (panel 9) were not prominent. The ventilatory reserve (panel 8) was not reached, but the level of performance, measured by the respiratory exchange rate, was sufficient.

Due to cardiocirculatory EC limitation in CPET, adenosine and/or dobutamine stress cardiac magnetic resonance imaging (cMRI) was performed after CPET1, to rule out myocardial pathologies, reduced left ventricular ejection fraction (LVEF), or underlying cardiocirculatory pathologies. Here, in sixteen patients cMRI was performed. None of these patients revealed a hemodynamically relevant ischemia, valve dysfunction, or LVEF reduction. While in thirteen patients the scan was completely unremarkable, one patient revealed focal, mid-ventricular to apical nonischemic myocardial contrast agent enhancement and another patient revealed a minimal basolateral edema. A third patient presented a pre-existing basoseptal contrast agent enhancement of unknown etiology. However, the significance of these changes remains unclear.

**Figure 2:**
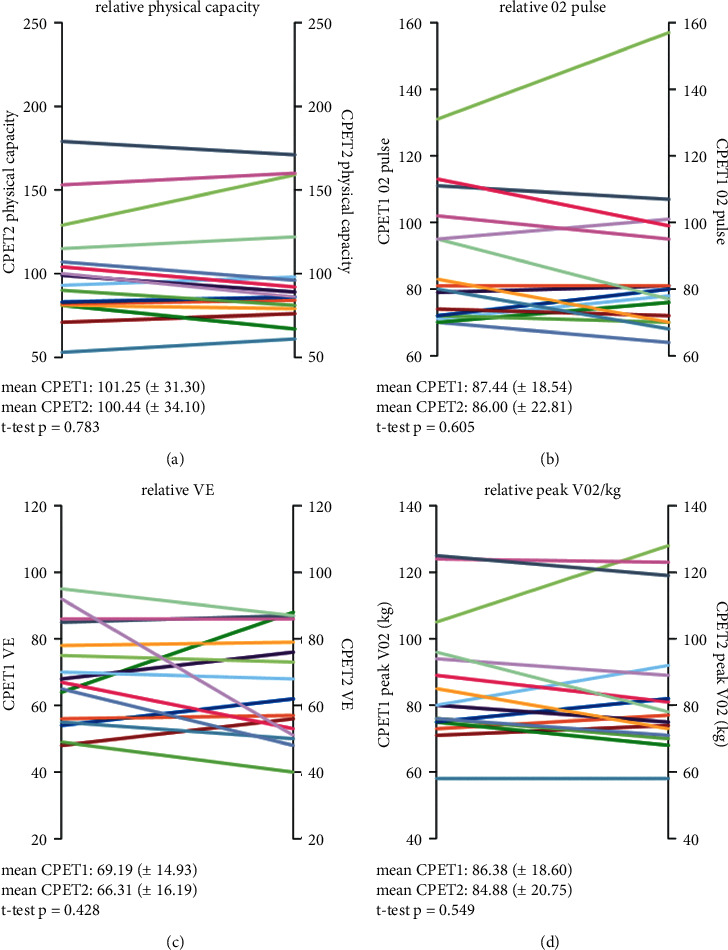
Physical capacity, O_2_ pulse, V̇E, and peak V̇O_2_/kg at first and second CPET (in %). Analysis performed with Student's *t*-test for related variables; mean value (±standard deviation). V̇E: respiratory minute volume and peak V̇O_2_/kg: body weight-indexed oxygen uptake.

Follow-up CPET (CPET2) was performed in objectively limited patients (*n* = 11), in patients with limited oxygen pulse (*n* = 10), and/or in patients with extensive subjective exercise limitation (*n* = 4). Though offered, two patients with limited EC in CPET1 refused to perform CPET2. Three patients with EC ≥ 100% did not achieve a lower limit of normal in EC during the second CPET. With respect to O_2_ pulse, follow-up CPET revealed limited O_2_ pulse in 10/16 (62.5%) workload-limited participants (Fisher's exact test *p*=0.008, data not shown). However, the deteriorated parameters of O_2_ pulse (*p*=0.605), peak V̇O_2_/kg (*p*=0.549), and V̇E (*p*=0.428) in our objectively limited subcohort neither improved nor declined over a period of 2.9 months from first to second CPET analysis (*c.f.*Figure 2) despite increase in physical activity.

## 4. Discussion

Cardiopulmonary exercise testing (CPET) is considered the gold standard for determining the degree of physical activity impairment [22, 25]. To the best of our knowledge, we are first in presenting data on sequential CPET in a patient cohort suffering from symptoms consistent with postacute COVID-19 syndrome. The majority of reports of health-related events following COVID-19 have been described after severe infection or hospitalization due to SARS-CoV-2 infection. Of interest, the ongoing presence of long-lasting symptoms such as shortness of breath and fatigue has also been recently reported in a significant number of nonhospitalized patients [26]. Likewise, our observations also refer to 9 patients, who showed a mild disease course and did not require hospitalization.

In our patient cohort, a typical pattern of reduced peak V̇O_2_ and reduced O_2_ pulse was identified as a possible pathognomonic pattern in objectifiable EC reduction. With respect to cardiocirculatory limitations, oxygen pulse indicates the capability of oxygen consumption of all body tissues per heartbeat and thus is a function of stroke volume and oxygen extraction by the cells. None of our patients had a known history of concomitant cardiovascular disease. Yet, considering the relevant differential diagnosis of impaired O_2_ uptake, deterioration of left ventricular ejection fraction, valve dysfunction, or impaired myocardial perfusion were widely excluded by stress cardiac MRI (cMRI) scans. With additional respect to the mean age of 52 years in this cohort, we assume that a possible influence of cardiovascular disease on the impaired EC in our patient population can be largely excluded. Contrary to our cMRI data, recent data on 26 college athletes with mild or asymptomatic SARS-CoV-2 infection described features consistent with persisting myocardial inflammation or previous myocardial injury in 15% and 30.8% of participants, respectively [27]. However, these data were sampled early after positive testing for COVID-19, *i.e.,* subsequent to recommended quarantine (11–53 days), and thus can reasonably explain the different observations.

Possible causes of persistent myocardial damage in patients with COVID-19 include ischemic damage due to endotheliitis [28] or epicardial coronary artery disease, and myocarditis [29] and stress cardiomyopathy [30]. In addition, right heart strain [31, 32] and systemic inflammatory syndrome [33] may lead to myocardial sequelae. However, the contribution of each of these causes to myocardial damage and to the potential limitation of cardiovascular exercise capacity in this context remains to be elucidated. Furthermore, it is reasonable to speculate that cardiac sequelae are more likely to occur in elderly patients and patients with severe COVID-19 disease, both of which are not true for the majority of our patients.

Acute COVID-19 is known to cause impairment of the circulatory system, with endothelial cell damage and vascular occlusion being the major contributors leading to hypoxemia [34, 35]. In this context, SARS-CoV-2 was shown to induce vascular endothelial cell dysfunction with SARS-CoV-2 spike protein leading to ACE2 downregulation [36] and impaired mitochondrial function [37]. Moreover, mitochondrial damage might play a relevant role in COVID-19 pathogenesis, as SARS-CoV-2 interacts via mitochondrial antiviral signaling protein (MAVS), finally impairing type I interferon production and leading to reduced mitochondrial oxygen sensing, oxidative stress-associated thrombocyte dysfunction, and induction of hemostatic pathways [38]. Other than that, Rovas et al. analyzed the vascular density, red blood cell velocity, and glycocalyx dimensions in tongue base microvessels via intravital microscopy by sidestream dark-field imaging in healthy individuals, nonventilated and ventilated COVID-19 patients [39], allowing for conclusions of graduated microvascular dysfunction with regard to COVID-19 severity. Additionally, microvascular changes in the retina were still detected in patients after bilateral SARS-CoV-2 pneumonia at 6 months [40] suggesting the possibility of persistent vascular impairment following COVID-19. Moreover, after COVID-19, persistent alterations of erythrocytes and neutrophils were most recently described [41]. Kubánková et al. hypothesize that the persisting changes in blood cell physical phenotypes could contribute to the long-term impairment of circulation and peripheral oxygen delivery [41].

Interestingly, chronic fatigue syndrome (CFS), a debilitating disease also caused by viral infections [42], shows similar features to long COVID syndrome, such as exertional intolerance with postexertional malaise and chronic fatigue. In CFS, vascular dysfunction leading to impaired muscle perfusion and limited cerebral blood flow upon exertion is considered a key mechanism for symptomatic disease [43–46]. In addition, mitochondrial dysfunction has been considered in CFS [47, 48]. In this regard, previous studies with CPET demonstrated reduced peak oxygen consumption in the majority of CFS patients [49, 50], although peak V̇O_2_ was not attributed to reduced oxygen uptake and transport to the muscle [51]. Since we were able to exclude a cardiac genesis as the cause of limited oxygen pulse and peak V̇O_2_, we hypothesize, based on our observations, that persistent vascular dysfunction with reduced peripheral oxygen delivery and/or impaired peripheral oxygen consumption due to metabolic dysfunction may also be present in our patient collective.

It seems questionable to what extent persistent symptoms in patients with a mild course of COVID-19 can be explained by pulmonary or cardiac dysfunction at all. Here, we did not find any evidence of persistent pulmonary or cardiac impairment in our patient collective. In addition, we could not demonstrate evidence of pulmonary vascular dysfunction in any of our patients, although CPET is considered a very sensitive method to detect pulmonary perfusion deficits. Pulmonary vascular sequelae such as thromboembolism are considered a major cause of severe COVID-19 disease [52–56]. Nonetheless, thromboembolic complications have also been described in single case reports in patients with mild disease [57–59]. Our patient collective included mainly patients with a noncritical disease course, so persistent pulmonary vascular dysfunction seems rather unlikely considering current scientific evidence. The extent to which chronic pulmonary vascular changes play a role in patients with long COVID syndrome and the diagnostic value of CPET in this context remains unclear.

Noteworthy, over an average period of 2.9 months from first to second CPET analysis neither EC, O_2_ pulse, nor oxygen uptake capacity (peak V̇O_2_/kg) significantly improved in our cohort (all *p* > 0.05). Hence, recovery after initial confounding physical detraining associated with severe COVID-19 is unlikely. A relevant limitation of the study is the missing patient's CPET data before COVID-19 manifestation. Therefore, EC limitations can only be diagnosed by comparison with healthy individuals or lower limit of normal values but do not consider a diverging individual physical fitness before infection with SARS-CoV-2. In this context, Milovancev et al. evaluated professional volleyball players three weeks after convalescence of COVID-19. While this cohort is thought to be well trained before SARS-CoV-2 infection, peak V̇O_2_/kg was deteriorated likewise in CPET [60], allowing for a concept of EC limitation pathogenesis after COVID-19 beyond pre-existing mitochondrial density and physical training.

The limitations of our manuscript are mainly due to the retrospective character of our data analysis. In addition, follow-up data were only available in a subset of patients, and there are no comparable baseline values to the performance status of patients before COVID-19. Furthermore, we did not systematically screen our patients for concomitant diseases and, therefore, cannot safely exclude an influence of cardiovascular sequelae on impaired physical performance status. This also applies to cardiac MRI examinations, which were not regularly performed in our patient cohort. Our statements on limited exercise capacity should, therefore, be interpreted with caution and rather be classified as hypothesis generating.

However, the major strength of our study is that we were the first to perform serial CPET examinations and our data clearly add information to the analyses of Wu et al. presenting a cohort of residual impaired patients with primarily reduced diffusion capacity of the lungs for carbon monoxide (DLCO) at month 12 after COVID-19 [5]. While in our cohort, gas exchange, measured by AaDO_2_, did not relevantly impair EC in limited patients, other factors such as reduced O_2_ pulse and reduced oxygen uptake capacity were prominent here, suggesting impaired peripheral oxygen metabolization.

## 5. Conclusions

Taken together, our investigation suggests a post-COVID-19-specific pattern of EC limitation. This limitation was primarily identified by a reduced O_2_ pulse, possibly due to reduced oxygen utilization and/or impaired peripheral oxygen metabolism in the absence of macroscopic cardiocirculatory pathology, occurring in at least a subset of patients with long COVID syndrome. Cardiopulmonary exercise testing may help identify this group of patients and thus could be considered for implementation in an expanded diagnostic work-up of long COVID syndrome. Further research is needed to fully understand the role of mitochondria, endothelial dysfunction, and peripheral oxygen utilization and to evaluate the role of CPET in this patient cohort. Therefore, larger cohorts of patients with long COVID syndrome need to be studied to further verify our observation.

## Figures and Tables

**Table 1:** Cohort characteristics.

#	Sex	Age (y)	CPET1										CPET2
			BMI (kg/m^2^)	FEV1 (%)	FVC (%)	COVID-19 severity	Time to CPET1 (m)	Workload max (%)	HF max (bpm)	O_2_ pulse max (%)	Peak V̇O_2_/kg (%)	RER max	
1	F	57	32	94	91	1	4.7	179	95	111	125	1.27	Y
2	M	76	26	76	81	2	3.9	107	151	70	76	1.16	Y
3	M	56	23	90	99	2	4.0	138	169	114	113	1.17	N
4	M	44	29	96	86	2	2.8	71	151	74	71	1.11	Y
5	M	57	29	95	87	3	3.5	81	157	70	75	1.20	Y
6	M	65	27	99	104	2	3.7	104	113	113	89	1.14	Y
7	M	57	26	96	83	2	3.6	115	157	95	96	1.19	Y
8	F	30	32	109	102	2	3.3	90	176	72	76	1.18	Y
9	M	63	21	104	99	3	3.2	129	137	131	105	1.17	Y
10	F	54	25	86	84	1	3.6	99	164	79	80	1.25	Y
11	M	43	26	124	119	2	2.8	83	179	72	75	1.20	Y
12	F	30	20	91	103	1	2.8	116	179	113	103	1.02	N
13	F	61	24	82	84	2	1.9	128	129	110	90	1.18	N
14	M	50	29	110	95	2	3.9	93	173	71	80	1.12	Y
15	M	60	27	93	87	3	6.0	53	111	80	58	1.09	Y
16	F	53	24	118	120	2	6.4	129	164	104	103	1.20	N
17	M	57	25	98	101	2	5.7	125	171	101	107	1.20	N
18	M	23	29	94	108	1	5.7	81	179	83	85	1.00	Y
19	M	39	25	99	96	2	4.3	82	166	81	73	1.08	Y
20	M	38	24	82	89	2	2.7	100	181	95	94	1.09	Y
21	M	72	29	114	118	2	4.9	124	113	117	100	1.04	N
22	F	34	22	84	110	1	6.9	127	181	107	102	1.13	N
23	M	61	28	101	96	2	2.8	153	176	102	124	1.07	Y
24	M	53	33	97	91	2	2.3	90	157	68	79	1.18	N
25	F	32	52	98	126	1	4.0	146	153	99	112	1.06	N
26	Ff	45	29	88	81	1	1.3	72	139	83	70	1.20	N
27	M	42	25	92	143	2	7.8	134	179	113	115	1.19	N
28	F	82	24	147	130	2	3.6	122	114	98	81	1.00	N
29	F	54	19	80	80	1	8.6	211	171	101	99	1.17	N
30	F	57	26	106	103	1	8.7	199	157	134	135	1.07	N
**AVG**		**52**	**27**	**98**	**100**		**4.3**	**116**	**155**	**95**	**93**	**1.14**	

# = patient number; sex F = female, m = male; age at COVID-19 diagnosis (in years); BMI = body mass index (in kilogram per square meter); FEV1 = forced expiratory volume in 1 second (in %); FVC = forced vital capacity (in %); COVID-19 severity: 1 = mild/moderate = outpatient treatment, 2 = severe = inpatient treatment, oxygen supplementation, 3 = critical = treatment at intensive care unit; time to CPET1 = time since positive SARS-CoV-2 PCR test to first CPET (in months); workload max = maximum work load (in %); HF max = maximum heart frequency (in beats per minute); O_2_ pulse max = maximum oxygen pulse (in %); peak V̇O_2_/kg = body weight-indexed peak oxygen uptake (in %); RER = respiratory exchange ratio; CPET2 y = yes, *n* = no, AVG = mean average.

**Table 2:** Comparators of non-CPET values.

Variables	Overall cohort	In %	Nonlimited CPET1	In %	Limited CPET1	In %	*p* value
	*n* = 30		*n* = 19		*n* = 11		
Sex							0.442^a^
Female	12	40	9	47	3	27	
Male	18	60	10	53	8	73	
Age							0.050^b^
Mean (±SD)	52 (±14)		55 (±14)		45 (±11)		
BMI							0.263^b^
Mean (±SD)	27 (±6)		26 (±7)		28 (±3)		
Patient care							1.000^a^
Outpatient care	9	30	6	32	3	27	
Inpatient care	21	70	13	68	8	73	
COVID-19 severity							0.582^c^
Mild/moderate	9	30	6	32	3	27	
Severe	18	60	12	63	6	55	
Critical	3	10	1	5	2	18	
Ventilatory support							0.366^a^
Noninvasive vent.	2	7	1	5	1	9	
Invasive vent.	1	3	0	0	1	9	
mMRC dyspnea sc.							0.497^c^
mMRC 0	8	27	6	32	2	18	
mMRC 1	20	67	12	63	8	73	
mMRC 2	2	7	1	5	1	9	
mMRC 3-4	0	0	0	0	0	0	
FEV1							0.746^b^
Mean (±SD)	98 (±14)		98 (±16)		99 (±11)		
FVC							0.106^b^
Mean (±SD)	100 (±16)		103 (±17)		94 (±12)		

*p* values: a = Fisher's exact test, b = Student's *t*-test of unpaired values, *c* = Mann-Whitney *U*-test; mean (±SD) = mean ± standard deviation; mMRC = modified medical research council dyspnea scale; FEV1 = forced expiratory volume in one second; FVC = forced vital capacity.

**Table 3:** Comparators of CPET1 and CPET2 values.

Variables	Overall cohort	Nonlimited CPET	Limited CPET	*p* value
CPET 1	*n* = 30	*n* = 19	*n* = 11	
Heart rate (bpm)	155 (±25)	152 (±27)	159 (±20)	0.421
Max. systolic blood pressure (mmHg)	172 (±18)	176 (±16)	165 (±19)	0.147
Max. diastolic blood pressure (mmHg)	87 (±11)	90 (±10)	82 (±12)	0.076
Workload (watt)	169 (±61)	183 (±63)	145 (±51)	0.086
Workload (%)	116 (±37)	136 (±30)	81 (±13)	**<0.001**
Anaerobic threshold (watt)	97 (±41)	107 (±43)	79 (±31)	**0.049**
Max. O_2_ pulse (ml)	13 (±4)	14 (±4)	12 (±3)	0.135
Max. O_2_ pulse (%)	95 (±19)	107 (±14)	76 (±6)	**<0.001**
Max. V̇E (l/min)	83 (±30)	86 (±33)	78 (±24)	0.463
Max. V̇E (%)	71 (±30)	76 (±21)	61 (±9)	**0.009**
Peak V̇O_2_/kg (ml/min/kg)	24 (±7)	26 (±7)	21 (±5)	**0.047**
Peak V̇O_2_/kg (%)	93 (±19)	104 (±15)	75 (±7)	**<0.001**
BR (%)	30 (±18)	25 (±20)	39 (±9)	**0.010**
RER	1.14 (±0.07)	1.13 (±0.07)	1.15 (±0.07)	0.623
pO_2_ at rest (mmHg)	81 (±9)	80 (±7)	83 (±11)	0.452
pCO_2_ at rest (mmHg)	38 (±3)	38 (±3)	38 (±3)	0.974
pO_2_ at submax. load (mmHg)	79 (±11)	75 (±11)	85 (±10)	**0.006**
pCO_2_ at submax. load (mmHg)	35 (±4)	36 (±4)	34 (±4)	0.351
AaDO_2_ at submax. load (mmHg)	35 (±9)	38 (±8)	29 (±8)	**0.006**
Lactate at submax. load (mmol/l)	5.5 (±2.1)	5.5 (±2.4)	5.4 (±1.6)	0.887

CPET 2		n = 4	n = 12	
Heart rate (bpm)	158 (±20)	161 (±20)	156 (±21)	0.693
Max. systolic blood pressure (mmHg)	169 (±16)	176 (±9)	167 (±18)	0.226
Max. diastolic blood pressure (mmHg)	87 (±7)	89 (±1)	87 (±8)	0.320
Workload (watt)	171 (±61)	228 (±54)	152 (±52)	0.060
Workload (%)	100 (±34)	153 (±21)	83 (±11)	**0.005**
Anaerobic threshold (watt)	96 (±44)	142 (±54)	80 (±28)	0.104
Max. O_2_ pulse (ml)	13 (±4)	16 (±4)	13 (±4)	0.222
Max. O_2_ pulse (%)	86 (±23)	109 (±34)	78 (±12)	0.171
Max. V̇E (l/min)	85 (±23)	103 (±17)	79 (±29)	0.079
Max. V̇E (%)	66 (±16)	83 (±7)	61 (±14)	**0.001**
Peak V̇O_2_/kg (ml/min/kg)	23 (±7)	28 (±8)	22 (±6)	0.268
Peak V̇O_2_/kg (%)	85 (±21)	112 (±23)	76 (±9)	0.048
BR (%)	34 (±16)	17 (±7)	39 (±14)	**0.001**
RER	1.13 (±0.08)	1.12 (±0.08)	1.13 (±0.08)	0.875
pO_2_ at rest (mmHg)	81 (±6)	75 (±5)	83 (±6)	**0.049**
pCO_2_ at rest (mmHg)	38 (±3)	38 (±2)	39 (±3)	0.338
pO_2_ at submax. load (mmHg)	86 (±9)	80 (±6)	88 (±10)	0.075
pCO_2_ at submax. load (mmHg)	34 (±5)	32 (±1)	35 (±6)	0.084
AaDO_2_ at submax. load (mmHg)	29 (±8)	35 (±12)	27 (±5)	0.235
Lactate at submax. load (mmol/l)	5.9 (±2.1)	6.2 (±0.3)	5.8 (±2.4)	0.521

Bpm = beats per minute; mmHg = millimeter mercury column; ml = milliliter; V̇E = respiratory minute volume; peak V̇O_2_/kg = body weight-indexed peak oxygen uptake; BR = breathing reserve; RER = respiratory exchange ratio; pO_2_ = oxygen partial pressure in capillary blood gas analysis; pCO_2_ = carbon dioxide partial pressure in capillary blood gas analysis; AaDO_2_ = alveolar-arterial oxygen gradient; mmol/l = millimole per liter.

## Data Availability

The datasets used and/or analyzed during the current study are available from the corresponding author on reasonable request.
